# 829. Incidence of Low BMD and Barriers to Routine Screening for Osteoporosis in HIV Patients in Eastern North Carolina

**DOI:** 10.1093/ofid/ofab466.1025

**Published:** 2021-12-04

**Authors:** Smit Rajput, Dora Lebron, Alicia Lagasca, Jaffer Hussain, Ogheneruona Odili, Suzy Nichols

**Affiliations:** 1 East Carolina University, Greenville, North Carolina; 2 ECU, Greenville, North Carolina

## Abstract

**Background:**

With HIV therapy, the life expectancy of persons with HIV (PWH) has improved and complications associated with long-standing HIV and antiretroviral drugs have become more apparent. Low bone mineral density (BMD) (defined by T score < -1) and osteoporosis (defined by T-score < -2.5) are common in PWH. In a meta-analysis of 884 HIV-infected patients, 67% had reduced BMD, of whom 15% had osteoporosis which is 3 times greater than HIV uninfected controls. IDSA guidelines recommend routine screening for osteoporosis in PWH aged ≥ 50 years, yet the rate of screening for osteoporosis in these patients remains low (7.4%-17%). This QI project aimed to estimate the frequency of and identify the barriers to screening for osteoporosis in eligible HIV patients.

**Methods:**

This prospective observational study was conducted in the HIV clinic at East Carolina University from 2018-2019. A sample of 104 HIV patients, ≥ 50 years were selected randomly. Data regarding referral for DXA (dual X-ray absorptiometry) scan, its results, and their insurance provider was collected. The plan was to analyze the barriers associated with guideline-recommended BMD screening and implement it in eligible patients.

**Results:**

From a total of 104, 89 patients (85.6%) were referred for a DXA scan. The reasons for lack of referral were obesity, insurance barrier, wheelchair-bound, and test ordered by another provider. Of the 89 patients referred for DXA, only 49 (47% of total) underwent the scan. In terms of barriers, insurance limitation was the most common reason. Out of the patients that had DXA scans, 19 (39%) were found to have low bone density and 1 had osteoporosis. Low BMD was more common in men (63%) as compared to women (37%) in this group.

Percentage of patients who underwent a DXA scan and the barriers in those who didn’t

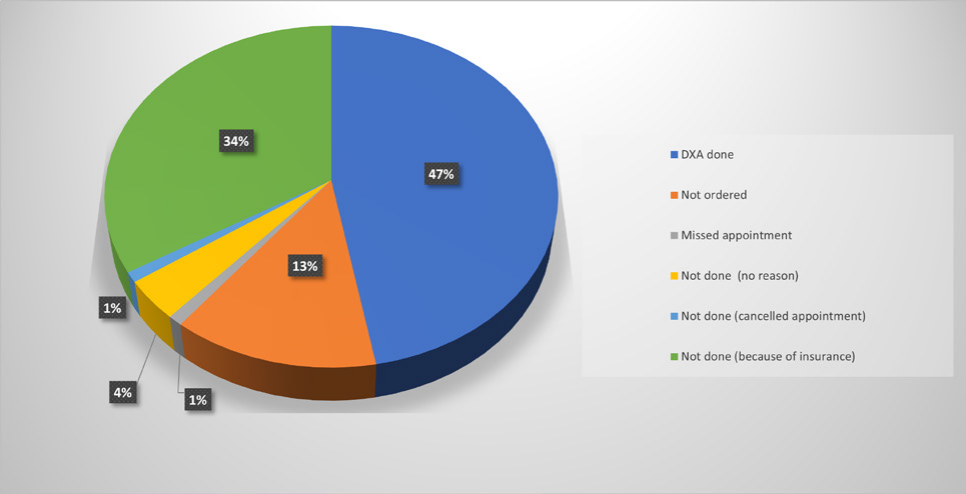

Frequency of BMD screening

Incidence of Low BMD

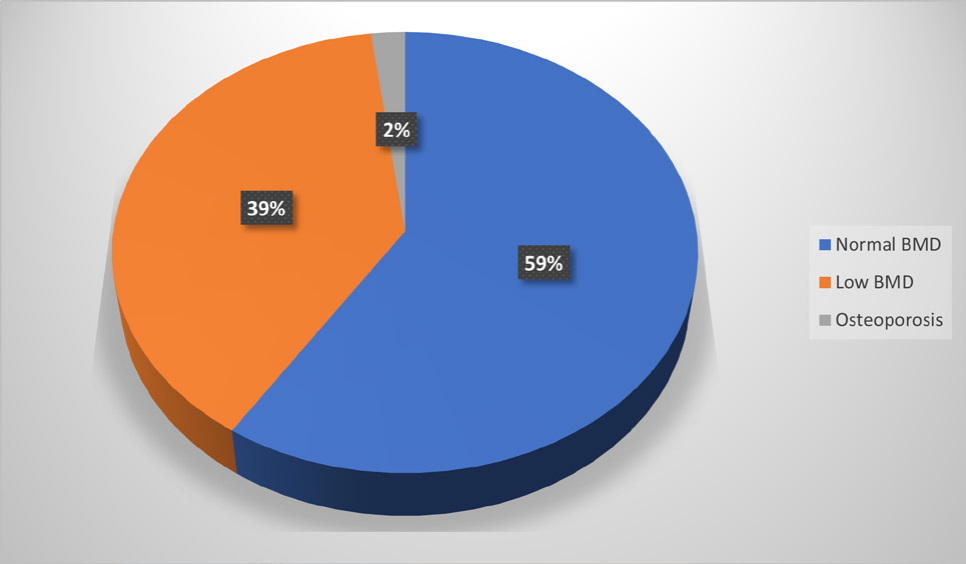

BMD results

**Conclusion:**

In our study, 47% of patients had a BMD assessment. This is better than what has been reported in other single-center studies, however, it is not ideal. About 34% of the patients had insurance coverage as the major barrier for routine screening, as has been mentioned in other similar studies. Of the patients who underwent the DXA scan, 41 % had a low BMD. Other studies have reported variable prevalence of abnormal BMD, from 47-93%. Interestingly, the prevalence of low BMD in our cohort was close to the national average in non-HIV patients.

**Disclosures:**

**All Authors**: No reported disclosures

